# Sudden cardiac death risk in the early vulnerable phase of heart failure: clarifying the SCD-PROTECT findings

**DOI:** 10.1093/eurheartj/ehag054

**Published:** 2026-02-13

**Authors:** David Duncker, Eloi Marijon, Johann Bauersachs

**Affiliations:** Hannover Heart Rhythm Center, Department of Cardiology and Angiology, Hannover Medical School, Carl-Neuberg-Str. 1, Hannover 30625, Germany; Division of Cardiology, European Georges Pompidou Hospital, Paris, France; Université Paris Cité, INSERM U970, Paris Cardiovascular Research Centre, Paris, France; Hannover Heart Rhythm Center, Department of Cardiology and Angiology, Hannover Medical School, Carl-Neuberg-Str. 1, Hannover 30625, Germany

**This commentary refers to ‘Sudden cardiac death in newly diagnosed non-ischaemic or ischaemic cardiomyopathy assessed with a wearable cardioverter-defibrillator: the German nationwide SCD-PROTECT study’, by D. Duncker *et al.*, https://doi.org/10.1093/eurheartj/ehaf668 and the discussion piece ‘The WCD dilemma: quantifying risk and appropriateness in the GDMT era’, by K. Gupta and M.K. Lahiri, https://doi.org/10.1093/eurheartj/ehag053.**

Dear colleagues,

Thank you for your excellent and viable questions.

Left ventricular ejection fraction (LVEF) measurement before index hospital admission is admittedly a rather vague value. Still, we gathered it to get all possible information. Importantly, a reduced LVEF at a single prior time point does not necessarily imply established chronic heart failure or a manifest cardiomyopathy. In many patients, LVEF deterioration occurred in the context of an acute or subacute clinical condition, ultimately leading to a new diagnosis of heart failure with a substantial drop in LVEF, subsequent hospital admission, and prescription of a wearable cardioverter-defibrillator (WCD). Patients with known chronic cardiomyopathy and persistently reduced LVEF would, in most cases, already have been evaluated for implantable cardioverter-defibrillator (ICD) therapy and therefore would not have been eligible for WCD prescription.

Your comment regarding the comparison with the meta-analysis by Shen *et al*.^1^ raises several important points that merit separate consideration. Beyond methodological concerns regarding the interpretation of declining sudden cardiac death (SCD) rates in that meta-analysis (as discussed elsewhere^2^), the populations and clinical phases addressed by Shen *et al*.^1^ and by SCD-PROTECT^3^ are fundamentally different. All trials included in the Shen meta-analysis enrolled patients with stable, chronic heart failure who were already established on medical therapy. By protocol design, these studies therefore could not capture the early, vulnerable phase following new heart failure diagnosis or recent clinical deterioration.

In contrast, SCD-PROTECT specifically assessed the early phase after hospital discharge, during initiation or early optimization of guideline-directed medical therapy (GDMT). In our cohort, the risk of sudden cardiac arrest (SCA) was highest in the first days to weeks after discharge, a period that is systematically missed in chronic heart failure trials. Consequently, direct comparison of cumulative event rates between these fundamentally different phases of the patient pathway is inherently limited.

You correctly note that SCD-PROTECT reports appropriately treated SCA, whereas randomized drug trials adjudicated SCD. However, it should be acknowledged that definitions and adjudication of SCD vary considerably across trials, and reliable identification of SCD remains challenging, even with autopsy data.^4,5^ Importantly, the WCD incorporates patient response buttons that allow conscious patients to withhold therapy. In addition, the delay from arrhythmia detection to treatment delivery, approximately one minute in the absence of patient response, allows for potential spontaneous termination of ventricular arrhythmias.^6^ Accordingly, appropriate WCD treatments can be reasonably considered surrogates of hemodynamically unstable VT/VF and thus clinically equivalent to aborted SCD. This concept differs from appropriate ICD shocks, which are known not to uniformly represent life-threatening arrhythmias.

With respect to event rates, the cumulative incidence of SCA in SCD-PROTECT was indeed 1.3%. While this may be juxtaposed with the 90-day cumulative incidences reported by Shen *et al*.^1^ (ranging from 2.4% to 1.0%), such a comparison neglects the markedly different follow-up durations across studies (∼60 days in SCD-PROTECT vs median or mean follow-up durations of 24–27 months in trials such as PARADIGM-HF or RALES). For meaningful comparison across heterogeneous follow-up periods, incidence rates expressed per 100 patient-years represent the appropriate and established metric. In SCD-PROTECT, the incidence rate of first appropriate treatment was 7.14 per 100 patient-years, which is comparable to -and at the higher end of the annualized rates (6.5% to 3.3%) reported in the Shen *et al*.^1^ meta-analysis.

Lastly, we appreciate your suggestion to report inappropriate WCD treatments using the same person-time format as appropriate treatments. While this is a valid methodological point, the primary endpoint of SCD-PROTECT was predefined as the incidence rate of appropriate treatments per 100 patient-years. Inappropriate treatments were rare and occurred at a very low frequency, and therefore do not materially alter the interpretation of the appropriate treatment rates or the overall risk–benefit profile observed in this cohort.

We again thank you for your insightful comments and the opportunity to further clarify these important aspects of the study.
